# Aspiration and Steroid Injection-An Effective Approach for Auricular Seroma 

**Published:** 2019-09

**Authors:** Sheetal Rai, Deviprasad Shetty

**Affiliations:** 1 *Department of ENT, Yenepoya Medical College, Mangalore city, Karnataka State, India.*

**Keywords:** Auricular Pseudocyst, Auricular Seroma, Aspiration, Intralesional steroid injection

## Abstract

**Introduction:**

Auricular seroma is a benign condition of the pinna usually following blunt trauma. This condition which presents with a simple swelling of the pinna is occasionally associated with pain and may result in permanent disfigurement of the pinna owing to delay in diagnosis or mismanagement. Various techniques have been proposed and practiced over the years to treat this uncomplicated condition. However, since this condition is notorious for its recurrence, it has always posed a challenge to the ear, nose, and throat surgeons. Therefore, a simple technique known as aspiration and intralesional steroid injection was proposed in this study for the treatment of auricular seroma.

**Materials and Methods:**

A total of 30 patients with a clinical diagnosis of auricular seroma were studied over a period of six years at a tertiary care hospital in Mangalore, India. The seroma was aspirated with a 22 gauge needle followed by intralesional injection of Triamcinolone acetate (40 mg/1 ml). The patients were followed up strictly for two weeks, one, three, and six months, as well as one year, and thereafter at yearly intervals as long as possible. No recurrence was observed as the main outcome of treatment for at least one year.

**Results:**

None of our patients had recurrence at the end of one year. In total, 15 patients followed up for at least two years. In addition, four patients are continuing follow-ups at the moment (i.e. six years post-treatment).

**Conclusion:**

Aspiration and intralesional steroid injection is a simple, minimally invasive, cost-effective, and a promising treatment modality which avoids recurrence.

## Introduction

Auricular pseudocyst also known as auricular seroma is a rare benign condition of the pinna usually following blunt trauma. It is caused by an intracartilaginous collection of serous fluid in the anterior aspect of the auricle most commonly in the scaphoid fossa. It presents as a simple swelling of the pinna, occasionally associated with pain and may result in permanent disfigurement of the pinna owing to delay in diagnosis or mismanagement. Various techniques have been proposed and practiced over the years to treat this uncomplicated disease. 

However, the commonly used technique is surgery which includes an incision and drainage followed by the excision of a piece of auricular cartilage using cartilage window technique and contour dressing. The surgical procedure warrants a hospital stay and is required to be performed under strict aseptic conditions and the administration of intravenous antibiotics, thereby adding to the financial burden imposed on the patient.

 This condition is notorious for its recurrence; therefore, it requires frequent revision procedures. Moreover, there are no long-term follow-ups available in the literature for the commonly practiced procedures. Accordingly, it is essential to seek better treatment alternatives and evaluate their long-term outcomes. With this background in mind, this study aimed to investigate the effectiveness of a simple and easy technique which is known as aspiration and intralesional steroid injection on the management of auricular seroma.

## Materials and Methods

A total of 30 patients were studied over a period of six years from October 2011 to 2017 at Yenepoya Medical College Hospital in Mangalore, Karnataka, India, after obtaining ethics approval. Relevant clinical data were obtained from the patients using demographic information form covering such information as age, gender, place of residence, occupational level, and medical history. Moreover, a detailed clinical examination was performed to examine the site and size of the swelling as well as associated findings. 

The patients were counselled regarding the procedure and the complications, such as the possibility of recurrence, and therefore, they were asked to participate in post-procedure long-term follow-ups. The patients who were willing to participate in the study were selected to enrol in the research procedure. 

The procedure was performed under strict aseptic precautions without local anaesthesia. A 22 gauge needle was used with a 5 cc syringe to aspirate the swelling at its most dependent part. The amount and colour of the aspirated fluid were recorded in this study. The needle was left in place and the syringe detached from the needle to prevent the cyst from collapsing completely. Subsequently, 0.1 to 0.2 ml of Triamcinolone acetate (40mg/1 ml) was injected through the same needle until it produced a bulge and the needle was withdrawn with a minimal leakage from the injection site (less than 0.05 ml). 

No pressure was applied locally and no antibiotic treatment was given following the procedure. The patients were asked to report immediately if the swelling increased in size or developed pain over the site. In addition, they were followed-up strictly at two weeks, one, three, and six months, as well as one year, and thereafter at yearly intervals till they were lost to follow-up.

## Results

A total of 30 patients with a clinical diagnosis of auricular seroma were included in this study. The majority of patients (93.3%) were males and they were within the age range from 20 to 50 years. The condition was more common on the right side (53.3%). In total, 16 and 14 patients presented with swelling on the right and left sides, respectively. Moreover, 18 patients (60%) complained of a painless swelling as their main complaint while the others (40%) presented with a mild pain associated with swelling. The majority of patients (53.33%) developed the swelling following some forms of blunt trauma and 12 cases (40%) developed the swelling spontaneously. Two patients (6.67%) referred with recurrence of swelling after the previous treatment - aspiration followed by pressure dressing in one case and incision and drainage in another (Table.1). 

Table 2 summarizes the most common sites of the swelling in this study including the conchal bowl (Cavum concha) followed by scaphoid fossa and cymba conchae. The amount of aspirated fluid ranged from 1 to 5 ml with an average of 2.74 ml. All patients reported a gradual increase in the swelling at the injection site which reached its maximum size on the fourth and fifth days, and thereafter reduced and disappeared completely by the end of the first week. None of the patients complained of pain, pigmentation, skin thickening, or itching even the two patients with type 2 diabetes mellitus.

Moreover, no recurrence was observed at the end of one year, and 15 patients followed up for at least two years. It should be noted that four patients are still continuing the follow-ups (i.e. six years post-treatment).

**Table 1:** Aetiology of auricular pseudocyst

**Cause**	**Number of patients**	**Percentage**
Blunt trauma	16	53.33%
Unknown	12	40%
Recurrence	2	6.67%
Total	30	100%

**Table 2:** Common sites of auricular pseudocyst

**Site**	**No. of cases**	**Percentage of cases**
Cavum concha	16	53.33%
Scaphoid fossa	10	33.33%
Cymba conchae	4	13.33%
Total	30	100%

## Discussion

Although auricular pseudocyst is rare and benign, it has always been a challenge to the clinician owing to its propensity to recur. In the present study, this condition was predominantly observed in males as supported by several other studies conducted by Tan et al. (1) and Lim et al. (2). According to the literature, the strikingly high prevalence in males was due to the differential actions of estrogen and testosterone in inducing cytokines, mainly IL-1 (3). The IL-1 is an important mediator of inflammation and cartilage destruction. It also induces IL-6 which stimulates chondrocyte proliferation. The analysis of the fluid from the auricular seroma for cytokine profile indicated markedly elevated levels of IL-6 and serum lactic dehydrogenase (LDH) values which support the traumatic etiology (4). 

The LDH-4 and LDH-5 have been proposed as major components of human auricular cartilage and are released following cartilage degeneration from repeated minor trauma, such as rubbing, ear pulling, sleeping on hard pillows, or wearing a motorcycle helmet or earphones. In this study, the most common site of the involvement was found to be the cavum concha contrary to other studies where scaphoid fossa has been described as the most common site of pseudocyst (3). 

Various surgical and nonsurgical modalities have been proposed for the treatment of this condition. In a study conducted by Sangeetha R. and Vijayendra H. in 2004, the cartilage was exposed by making a linear incision, and the rectangular piece of cartilage was removed closing the wound with a drain in situ. They found no recurrence or deformity after two months of follow-up (5).

Several non-surgical treatment modalities have been proposed, such as simple aspiration, aspiration and compression dressing, aspiration with intralesional steroid and oral steroid, aspiration and pressure dressing by a plaster of Paris cast (6-8). In a study performed by S K Pani et al., they compared the treatment modalities for pseudocyst of the pinna and followed up their patients up to 3 months. According to the results, aseptic aspiration followed by compression buttoning and surgical deroofing followed by compression buttoning were both equally effective procedures, with the former being more advantageous in terms of cosmesis, less time consuming, and cost-effectiveness (9). 

A study similar to the current one was conducted by Kim et al. in which intralesional steroid injection was utilized for recurrent auricular pseudocyst followed by clip compression dressing (10). The disadvantage of local positive pressure application by methods, such as contour dressing, plaster of Paris cast application, clipping, and buttoning is that they cause discomfort along with pain, and they occasionally lead to ischaemic skin necrosis (7,11). 

In the same line, Karthikeyan DA et al. performed a study in which aspiration and steroid injection was followed by negative suction pressure application using a 20 cc syringe and an external dressing which were removed after 24 hours. The patients were given antibiotic treatment and followed up to three weeks. The procedure was repeated in case of any collection. Despite the presence of no complication or recurrence in this study, the syringe hanging beside the ear in the initial 24 h led to patient discomfort (12).

 In a recent study conducted by Dabholkar Y et al., the pseudocyst was completely aspirated, and the compression dressing was applied for 2 weeks employing a silicone-based material that is used for making hearing aids. They found complete resolution of the swelling without any signs of recurrence even during a six-month follow-up. There was one case of recurrence requiring a repeat procedure. Though effective, the major disadvantage of the procedure was that the patient would have to bear the compression dressing for 2 weeks which added to the discomfort and also gave an unsightly appearance (13). In the present study, the procedure of aspiration and steroid injection was performed with aseptic precautions. A 22 gauge needle was employed to reduce the pain associated with the needle prick. The needle was retained in place after aspiration and the same was used for steroid infiltration to avoid a second prick. This also prevented the space from collapsing completely while making room for the steroid to accumulate within the subperichondrial space. The patients did not require analgesics after the procedure. Moreover, no local pressure and antibiotic treatment were given to the patients in this study. In addition, they did not require any external dressings. No post-procedure incidence of perichondritis or secondary skin changes were observed even in two patients with diabetes mellitus. The deformity of the pinna which is of prime concern in a surgical technique can be avoided by this simple approach.

Although there are so many treatment alternatives, the high chances of recurrence make patients reluctant to opt for surgical options. Therefore, the patient noncompliance resulted in poor follow-ups which led to inadequate literature support regarding long-term benefits of treatment modalities. The majority of studies have patient follow-ups for only two to three months. Therefore, this study adopted a simple, minimally invasive, and cost-effective technique. Patient compliance was good not only in terms of accepting the treatment option but also in regular follow-ups.

Furthermore, none of the patients had recurrence at the end of one year, and 50% of them (n=15) followed up for at least two years. Currently four patients are continuing the follow-ups (i.e. six years post-treatment). No patient complained of pain, pigmentation, skin thickening, or itching even the two patients who were suffering from type 2 diabetes mellitus.

Moreover, the technique was proven useful even in two patients after the recurrence following another primary procedure. One of the patients had undergone aspiration and pressure dressing as the primary procedure while the other received incision and drainage. 

## Conclusion

In this study, aspiration and intralesional steroid injection was proposed as a simple and minimally invasive procedure which is cost-effective and patient-friendly. It is a promising treatment modality without any complications and risk of recurrence.
